# Scaling up health technology assessment capacities in selected African countries – A conceivable route ahead

**DOI:** 10.1017/S0266462323000016

**Published:** 2023-01-30

**Authors:** Debjani Mueller, Leila Alouane, Mouna Jameleddine, Irene Lenoir-Wijnkoop

**Affiliations:** 1 School of Health Systems and Public Health, University of Pretoria, Pretoria, South Africa; 2 Independent Consultant in Nutrition, Tunis, Tunisia; 3 Health Technology Assessment Department, National Authority for Assessment and Accreditation in Healthcare (INEAS), Tunis, Tunisia; 4 Department of Pharmaceutical Sciences, Utrecht University, Utrecht, The Netherlands

**Keywords:** Assessment capacities, Health Technology Assessment, Malnutrition, Nutritional care, multi-country study

## Abstract

**Background:**

This study aimed to provide a structured description of the commonalities and differences in healthcare structures across Africa to establish a reliable basis for the health technology assessment (HTA) of nutrition and nutrition interventions. A ranking of current nutrition conditions in the general population of the participating countries was included to gain a better understanding of the factors influencing hospital malnutrition (HMN), which will inform future multi-country research.

**Method:**

A questionnaire on the structure of the health systems was distributed among ten African countries. Subsections were included that inquired about the drivers or barriers to using principles of HTA to assess nutritional care. Analysis and ranking of malnutrition data were based on data from the Global Hunger Index report and two poverty indicators used by the World Bank.

**Results:**

The health system structure of each country was identified and described, whereas questions about HTA could not always be analyzed due to a lack of adequate in-depth knowledge and skills in most countries. Early experience from some countries demonstrates a conceivable route ahead for African countries in strengthening the capacity for and implementing HTA in accordance with distinct national healthcare contexts and social determinants of health.

**Conclusion:**

Problems related to nutritional care represent one of the major priorities in the surveyed countries. A future HMN multi-country study will provide valuable insight into the potential of low-cost primary prevention orientations.

## Introduction

It is a well-known fact that the sustainability of health systems is enhanced by investing in disease prevention, health promotion, and early diagnosis instead of treatment (1). As the limits of curative medicine become apparent and the cost of medical care escalates in all countries, the interest in nutrition-oriented interventions as adjuvants in disease management has increased over the past decade and an impressive number of studies confirm the clinical relevance and cost-effectiveness of nutritional support in the hospital setting (2). Nutrition interventions have the benefit of being low-cost, and screening tools for assessing the nutritional status of a subject are readily available in any region, as are healthcare providers with the capacity to apply them. Despite this, the application of health technology assessment (HTA) in the field of nutrition is still very limited; existing HTA studies are limited to high-income countries and do not address a broader approach to the consequences of adequate nutrition on health care (3). Decisions about nutritional support modalities take place predominantly in a multidisciplinary framework (4) and, as a result, are an ideal candidate for evaluation using the comprehensive methods of integrated HTA.

The implementation of the HTA process enables the evaluation of existing health systems and finding solutions to the factors that hinder the progress of universal health coverage (UHC); a context that has led to an expanding need for a number of HTA agencies and/or capacities worldwide (5). The WHO Global Survey on HTA carried out among the 194 member states, reported that from the six WHO regions, African countries had the lowest response rate, possibly related to a lack of established systems for undertaking HTA (6). This is in accordance with a recent study showing that awareness of HTA and economic evaluation in Sub-Saharan countries is low, with inadequate expertise and a lack of local data and tools, due to – among other things – the (i) resource-limited environment and (ii) low or nonexisting political commitment (6;7). One way the first issue might be addressed, is by setting up a multi-country initiative, as this would allow mutualizing of the existing competences from several countries. Indeed, as expressed by Velasco Garrido et al. “*Countries embarking on HTA should not consider establishing separate agencies for HTA, […], but should rather combine these functions and goals into a common knowledge strategy for evidence-informed decision-making on health care and the health system*” (8). We hypothesize that the demonstration of cost-effective results of a low-cost intervention in a commonly occurring health concern would lead to an increasing interest from local instances in charge of healthcare resource management, and that quantified outcomes of better management of hospital malnutrition (HMN) will gradually encourage them in recruiting/training HTA skills, thus creating a virtuous circle. This would help to initiate a bottom-up process of routine data collection, for gradually building/scaling up an evidence base. The urgent need – shared by most African countries – to address the double burden of malnutrition might inform the selection of an appropriate health intervention (9). There are multiple forms of malnutrition and most of them are extremely difficult to assess in a reliable manner, because of complex interactive environments characterized by a high number of uncertainties. This hurdle can be circumvented by conducting health-economic evaluations in the hospital setting, which ensures meaningful outcomes for further exploitation as reported for other Low- or Middle-Income Country (LMIC) regions (2;10–12). Furthermore, when considering a future multi-country clinical protocol on HMN, it is crucial to identify commonalities and differences among national African healthcare systems.

## Objectives

The objectives of our study were twofold:

- Provide a brief description of the current nutrition-related health in African countries that influences malnutrition in general.
- Describe the commonalities and differences in national healthcare structures across Africa, in order to establish a reliable basis for a future multi-country clinical protocol on HMN.

## Methods

In collaboration with a medical doctor from Malawi (BC) and a nutritionist from South Africa (MT), a snowball sampling approach was used to recruit specialists in the area of public health and clinical nutrition from ten African countries (Cameroon, Ethiopia, Kenya, Malawi, Nigeria, Senegal, South Africa, Tanzania, Zimbabwe, and Tunisia).

For a better understanding of the factors that influence malnutrition, the current health conditions in the concerned countries were examined and ranked based on (i) the Global Hunger Index (GHI) (13), (ii) two poverty indicators used by the World Bank to estimate poverty (14), and (iii) the available life expectancy statistics.

In parallel, a questionnaire, based on a survey previously used for addressing the challenges of implementing a HTA Policy Framework in South Africa was adapted and distributed among the members of the multi-country study group who were asked to complete the questionnaire with information from their respective countries (Supplementary Table 1). The questionnaire was structured into three sections: (a) a main section to collect information on the structure of the country’s health systems, and two subsections to capture the role of HTA (if any) in the health system enquiring about (b) drivers or barriers for utilization of HTA within the health system and (c) challenges regarding the development of capacities in HTA. These survey indicators were selected with the aim to facilitate the implementation of the future multi-country study of HMN in Africa. Consideration was given to the existing infrastructure within the population, as it shows the peoples’ interest in their health.

In some cases, the participants needed to consult local experts to answer certain questions. These local experts were academics or collaborators from the country’s Ministry of Health. The responses were additionally validated with published and extensively referenced documents. The survey findings were compiled using a Microsoft Excel program, 2016, and the free text comments were analysed to determine the similarities and dissimilarities of the different health systems (full survey results are available in Supplementary Table 2).

## Results

The results of the analysis of the factors that cause malnutrition are presented first, followed by the results of the questionnaire.

### Conditions that influence malnutrition

#### The Global Hunger Index (GHI)

The GHI represents the worldwide evolution of hunger over time. The 2021 report (15) shows that hunger persists in many countries; Sub-Saharan Africa is the region with the highest GHI 2021 scores. The GHI evolved positively but unevenly between 2000 and 2021, for the ten countries. Only Tunisia reached a low severity scale, Senegal and South Africa reached a moderate severity scale, while the other seven countries so far have a serious severity scale. However, Ethiopia was able to move from an extremely alarming state (53.5 percent) to a moderate state of severity (24.1 percent) during the above-mentioned timespan. Zimbabwe seems to have difficulties enhancing its nutritional situation, staying at a very high level of hunger (34.9 percent) as shown in Table 1.

**Table 1.** Progression of Global Hunger Index (GHI) Scores from 2000 to 2021 and country rank according to the international classification of poverty indicators

				Country ranking		
Country	GHI 2000	GHI 2021	GHI change over 21 years (%)	GHI rank 2021^a,^ ^d^	Poverty headcount ratio at national poverty lines^b^	Poverty headcount ratio at USD 1.90 a day^c^
Cameroon	35.7	18.6	−47.9	74	42	37
Ethiopia	53.5	24.1	−55.0	90	74	31
Kenya	36.7	23.0	−37.3	87	44	28
Malawi	43.1	21.3	−50.6	81	20	4
Nigeria	39.5	28.3	−28.4	103	30	12
Senegal	34.0	16.3	−52.1	66	25	25
South Africa	18.1	12.9	−28.7	60	14	42
Tanzania	40.6	24.7	−39.2	92	58	18
Tunisia	10.3	6.0	−41.7	22	102	125
Zimbabwe	39.1	34.9	−10.7	4	3	40

a Ranking among 116 countries.

b Ranking among 131 countries (the classifications are older than the year 2021).

c Ranking among 141 countries (the classifications are older than the year 2021).

d Individual scores could not be calculated and rank could not be determined owing to lack of data. Zimbabwe hunger index was provisionally designated as alarming.

#### Poverty

The two indicators used by the World Bank to estimate poverty in the countries considered in this study are:

- Poverty headcount ratio at national poverty lines (percentage of the population): the percentage of the population living below the national poverty lines. National estimates are based on population-weighted subgroup estimates from household surveys (https://www.indexmundi.com/facts/indicators/SI.POV.NAHC).
- Poverty headcount ratio at USD 1.90 a day: the percentage of the population living on less than USD 1.90 a day at 2011 international prices (15).

In three of the participating countries, Zimbabwe, Nigeria, and Malawi, over 50 percent of the population lives below the poverty line. For Zimbabwe, this rate reached 70 percent in 2019, ranking it as the third poorest country in the world (Table 1) after Equatorial Guinea and Madagascar. The last report did not provide updated information on Zimbabwe. Only Tunisia is below 20 percent.

The classification compared to the poverty headcount ratio at USD 1.90 a day places Malawi at the top of the list. Internationally Malawi is ranked fourth after Madagascar, Congo, and Burundi. Tunisia has only 0.3 percent of people living below USD 1.90 a day.

Poverty amplifies the risk of, and risks from, malnutrition, leading to increased morbidity and mortality as well as higher health expenditures.

#### Life expectancy

Worldwide, people aged 65 and over represent 8.7 percent of the general population. This percentage is almost reached in Tunisia where the elderly represents 8.3 percent of the total population, with the longest life expectancy in Africa (76.31 years) after Algeria (76.5 years) in 2018. This rate is 5.3 percent for South Africa (life expectancy of 63.54 years). For the other countries, the average is 2.8 percent with 2.3 percent in Kenya and 3 percent in Ethiopia. Life expectancies range from 67.38 years in Senegal to 53.95 years in Nigeria (https://data.worldbank.org/indicator/SP.DYN.LE00.IN?locations=ZG). Global life expectancy is around 72 years. It has increased worldwide by an average of 21.4 years as shown in Table 2.

**Table 2.** Evolution of life expectancy at birth from 1960 to 2018

	Life expectancy at birth				Years gained since 1960	Healthy life expectancy^a^	Healthy life expectancy at age 60 (years)
	1960	1980	2000	2018		2016	2016
Cameroon	41.8	51.5	51.0	58.5	16.7	50.3	12.5
Ethiopia	38.4	43.8	51.9	65.9	27.5	56.1	13.5
Kenya	46.8	57.9	50.9	65.9	19.2	55.6	14.2
Malawi	36.7	44.3	45.1	63.3	26.6	51.2	12.8
Nigeria	37.0	45.3	46.3	54.0	17.0	47.7	10.7
Senegal	38.2	48.9	57.8	67.4	29.2	58.3	12.9
South Africa	48.4	58.1	56.1	63.5	15.1	55.3	12.5
Tanzania	43.6	50.3	50.8	64.5	20.9	54.1	13.4
Tunisia	42.0	62.0	73.2	76.3	34.3	66.7	15.1
Zimbabwe	53.0	59.7	44.7	60.8	7.8	52.3	13.4
World	52.6	62.8	67.5	72.6	20		

a https://apps.who.int/gho/data/view.main.HALEXv?lang=en.

Additionally, the number of doctors and paramedics and the number of beds per inhabitant are key indicators for the quality of health care. Only Tunisia and South Africa have figures close to the world average. For the other countries participating in this survey, the data relating to health professionals and the number of hospital beds do not all date from the same census year. Comparison between the countries should therefore be avoided as well as any interpretation against global figures as depicted in Table 3.

**Table 3.** Health workers and hospital beds per 1,000 people

Country	Physicians^a^	Nurses and midwives^b^	Hospital beds^c^
Cameroon	0.088 (2011)^d^	0.916 (2011)	1.3 (2010)
Ethiopia	0.077 (2018)	0.713 (2018)	0.3 (2016)
Kenya	0.157 (2018)	1.165 (2018)	1.4 (2010)
Malawi	0.036 (2018)	0.438 (2018)	1.3 (2011)
Nigeria	0.384 (2018)	1.179 (2018)	0.53 (2004)
Senegal	0.0691 (2017)	0.312 (2017)	0.33 (2008)
South Africa	0.9054 (2017)	1.307 (2017)	2.3 (2010)
Tanzania	0.014 (2016)	0.584 (2017)	0.7 (2010)
Tunisia	1.3025 (2017)	2.513 (2017)	2.1 (2017)
Zimbabwe	0.21 (2018)	1.934 (2018)	1.7 (2011)
World	1.563 (2017)	3.816 (2018)	2.9 (2017)

a Physicians include general and specialized medical practitioners.

b Nurses and midwives include professional nurses, professional midwives, auxiliary nurses, auxiliary midwives, enrolled nurses, enrolled midwives, and other associated.

c Hospital beds include inpatient beds available in public, private, general, and specialized hospitals and rehabilitation centers. In most cases, beds are for both acute and chronic patients.

d The numbers in brackets represent the census year.

### Responses from the questionnaire

All participating countries provided feedback on the survey questionnaire, in most cases based on informal data gathering. Cameroon, Ethiopia, Kenya, Nigeria, Senegal, South Africa, Zimbabwe, and Tunisia each had a single respondent, while Malawi and Tanzania each had two. While the structure of each country’s health system was generally well identified and clearly described, it appeared that the questions related to HTA could not always be analyzed due to the absence of application of HTA knowledge and skills to public health interventions such as preventive care in most countries.

#### Health systems organization

Healthcare systems can globally be divided into three types: centralized, decentralized, and mixed.

A major difference between these types lies in:

- The status of care providers.
- The type of social protection of the population.
- The method of financing the health system.

In our study, the countries that have adopted the centralized system are Cameroon, Malawi, Nigeria, and Zimbabwe. The other countries Ethiopia, Kenya, Senegal, Tanzania, and South Africa adhere to decentralized systems, while Tunisia has a mixed system.

#### The compulsory insurance scheme coverage

Health insurance coverage can be considered a strategy to improve access to care and a means of combating the impoverishment generated by potential catastrophic expenses. The World Health Organization (WHO) considers health insurance a promising means for achieving universal healthcare coverage (16). In Africa, 60 to 70 percent of health expenditure is directly paid by households (out-of-pocket expenditure), compared with an average of 46 percent worldwide. As shown in Table 4, the out-of-pocket expenditures for 2018 in the countries of our study, varied from 7.7 percent in South Africa to 76.6 percent in Nigeria with a tendency to decrease in all these countries except Nigeria, Senegal, and Tunisia where the rates increased between 2000 and 2018.

**Table 4.** Out of pocket expenditure as a share of current health expenditure (%)

Country	2000	2010	2018	Tendency between 2000 and 2018 (%)
Cameroon	77.4	71.7	75.6	−2.3
Ethiopia	35.9	42.3	35.5	−1.1
Kenya	47.1	30.2	23.6	−49.9
Malawi	17.3	10.9	11.2	−35.3
Nigeria	60.4	76.9	76.6	26.8
Senegal	55.6	48.7	55.9	0.5
South Africa	15.1	8.5	7.7	−49.0
Tunisia	38.6	42.1	38.9	0.8
Tanzania	38.5	31.9	23.9	−37.9
World	19.4	18.4	18.1	−6.7
Zimbabwe		33.8	24.3	−28

*Note*: For Zimbabwe, the tendency was calculated between 2010 and 2018 (last update April 2020).

https://knoema.fr/atlas/topics/Santé/Dépense-de-santé/Out-of-pocket-expenditure-as-a-share-of-current-health-expenditure.

Various types of health insurance are available:

- National or social health insurance (SHI) is based on individuals’ mandatory enrolment. Kenya, Nigeria, Senegal, Tanzania, and Tunisia are affiliated with SHI.
- Community-based health insurance (CBHI) scheme is an emerging alternative to increase primary healthcare access. CBHI is a form of micro-health insurance targeting low-income people utilized primarily in rural areas of developing countries. It is usually based on the following characteristics: voluntary membership, nonprofit objective, linked to a healthcare provider (often a local hospital), risk pooling, and reliance on a mutual aid/solidarity ethic (https://www.who.int/news-room/fact-sheets/detail/community-based-health-insurance-2020). Ethiopia, Kenya, Tanzania, and Senegal have been implementing the CBHI scheme. There is a growing interest in the role of the CBHI schemes given their impact on improving equity and access to essential health care for the poor, particularly informal sector workers (Supplementary Table 3).

#### Priority areas

Maternal and child health ranks high in the health priorities of all the countries covered by the questionnaire. Communicable diseases (tuberculosis, cholera, HIV/AIDS, and malaria) are a concern for all countries except Senegal and Tunisia. Communicable diseases are still rampant in many African countries as noted in the 2016 Annual Report of the Communicable Diseases Cluster of the WHO African Region (17). Although a primary prevention orientation is seldom present, nutritional problems, environmental hygiene, vaccination coverage and the fight and prevention against noncommunicable diseases (NCDs) are also priorities for the countries surveyed. The double burden of deficiency diseases is starting to become a major public health problem in countries like Tunisia, Senegal, South Africa, Malawi, Nigeria, and Kenya and is particularly acute in rapidly expanding urban areas and populations. In a recent study on the burden of NCDs in Sub-Saharan Africa, the results show that the all-age total of disability-adjusted life-years (DALYs) due to NCDs increased by 67 percent between 1990 and 2017 (18).

#### Health services delivery

Health facilities, providing curative and preventive services, are a central component of health systems. Access to health care is a complex and multidimensional concept, however, in its most narrow sense it refers to geographic availability. Defining the location of health services according to the communities they are intended to serve, is the cornerstone of health system planning. It ensures that the right services are accessible to the population and that no one is left behind or geographically marginalized from essential services.

For all the countries surveyed, the health system includes primary, secondary, and tertiary structures. In addition to these public structures which try to bring health services closer to the population, some countries have private structures (Tunisia, Malawi, and South Africa) too. According to the survey answers, in most cases, prevention does not seem to be a distinct part of health care and most services are curative. Some countries indicate preventive care, which probably is comparable to secondary or tertiary prevention only and often assimilated to primary care.

#### Hospitals’ health spending

The 2019 United Nations Economic Commission for Africa report notes that while several African governments have increased the part of the budget allocated to health, overall health financing remains a major obstacle to delivering effective health services. In addition, economic growth slowdown and high public debt have constrained the fiscal space for public financing of health care. Total health expenditure in Africa remained in a narrow band of 5 to 6 percent of GDP between 2000 and 2015 on average, although per capita, it almost doubled from USD 150 to USD 292 (in constant purchasing power parity [PPP] dollars). Due to the scarcity of public resources and the unpredictability of aid from donors, many people have been forced into poverty as a result of high private spending.

#### Remuneration of health service providers

Overall, remuneration differs across health professionals, scales, and levels; more detailed information about the survey answers on the remuneration modalities in the specific countries is available in Supplementary Table 2.

#### Organization of procurement

The subject of procurement is complex. Although the surveyed countries did not provide a detailed answer, they all referred to supply units located throughout the government. Distribution is often ensured by a joint procurement body; for Senegal and Tunisia, this structure also supplies the private sector.

#### Universal health coverage

According to estimates from a World Health Report on the path to universal coverage, 10 years ago, 20 percent to 40 percent of all health was wasted through inefficiency and the report described health financing mechanisms to ensure a more equitable coverage (16). UHC is based on the principle of health as a legal human right, through three coverage dimensions: population coverage, financial protection, and access to health services, aiming at equality of opportunity for people to enjoy the highest possible level of health. Over the last few years, the UHC movement has gained global momentum. In most countries in our survey, UHC is based on people-centered health care. It focuses on helping to improve well-being and quality of life with the assurance of equity between regions. The respondents from Nigeria, Zimbabwe, and Malawi did not specify the basis for UHC (Supplementary Table 2).

#### National patient organizations

Patient associations or organizations seem to be inexistent in Cameroon and Zimbabwe, rare in Ethiopia, Tanzania, and Kenya, and much more numerous in Malawi, Nigeria, Senegal, and South Africa. In Tunisia, the number of patient associations continues to increase, some have been present for over 40 years, and others are very recent. Unlike other countries where they primarily unite patients with transmissible or organic diseases, patient associations in Tunisia are mainly focused on NCDs, mental health, and various physical and mental disabilities.

#### Challenges related to HTA capacities

The respondents to the questionnaire confirm that the countries under study are at different stages in their national HTA institutionalization, with currently Tunisia being the only country with a national HTA entity. The other countries face numerous challenges including limited resources and expertise in HTA.

For Cameroon, the main challenge that needs to be addressed is the improvement of the training and working conditions of healthcare professionals and finding solutions for the lack of provision of adequate drugs and equipment. Developing HTA capacity and building regional collaborations for HTA institutionalization would enable the country to take advantage of the curricula changes occurring in most tertiary institutions in Africa. According to the respondents, an independent agency supported by an academic institution would be the most appropriate setup for the country.

One of the main challenges in the Ethiopian health system is the transition from communicable to NCDs which creates a double burden. It is necessary to assess this transition for enabling public health policies to obtain appropriate evidence on a larger scale. HTA has been implemented only in a fragmented manner in the Ethiopian health sector decision-making cycle, and the sector has been hampered by limited institutional capacity and skilled human resources to inform evidence-based decision-making. Currently, the Ethiopian Public Health Institute prepares evidence-based briefs. This governmental research institution is responsible for addressing and directing the national evidence needed for HTA.

Tunisia has been able to set up a central HTA body: The National Authority for Assessment and Accreditation in Healthcare INEAS. INEAS is the national scientific authority that informs decision-making particularly in relation to coverage of health technologies and interventions and aims to improve the quality of health care.

HTA is partially but not consistently applied in South Africa. Even though pharmacoeconomic guidelines are available, these are not widely used. The committee on the Essential Drug List of the National Department of Health (NDoH) considers certain aspects of HTA for their assessments. The Pricing Committee of the NDoH which regulates the private sector considers the therapeutic value of the medicine. The main challenges faced in the implementation of HTA or the use of evidence on decisions regarding technologies lie in the lack of competent knowledge and skills, political reluctance, limited technical expertise, corruption, and divided pricing structure and processes for state and private health care.

## Discussion

This study aimed to outline a conceivable route ahead for African countries for improving HTA awareness and federating/facilitating HTA capacity building by providing a structured description of the commonalities and differences in national healthcare structures across Africa, thus establishing a reliable basis for a future multi-country study. We briefly described the conditions of malnutrition in general and analyzed a questionnaire involving physicians, nutritionists, and HTA researchers from ten countries in Africa. The responses covered the current structure, organization, and priorities of the healthcare system in these countries.

These countries have either centralized or decentralized health systems and out-of-pocket expenditures on the national level vary significantly (7.7 percent to 76.6 percent). In addition, some countries provide a SHI scheme, while others apply CBHI schemes. Social protection programs such as that of the Food and Agriculture Organization (FAO) of the United Nations (19) are found to be effective tools to address both the drivers and the consequences of food insecurity and malnutrition, which may result in greater resilience in Africa.

A fundamental challenge for all health systems is to allocate limited resources to the unlimited demand for health services (20). It’s a rationing problem that requires priority choices. Inevitably, part of the demand is not met, which gives the impression of insufficient efforts attributed to health. Each country establishes its priority according to health system needs. In most cases, the priorities go toward the curative much more than toward the preventive aspects (20). In the developing part of the world where healthcare resources are even more limited than elsewhere, the role of HTA in guiding investment decisions is fundamental in order to recreate an optimal balance.

The World Health Assembly (WHA) Resolution WHA67.23 (21) on health intervention and technology assessment in support of UHC, urges the member states “*to consider establishing national systems of health intervention and technology assessment in support of universal health coverage to inform policy decisions….as well as formulation of protocols for public health programmes.”* Furthermore, the WHA expresses concern that “the *capacity to assess, research and documents public health, … implications of health interventions and technologies is inadequate in most developing countries.”* This study is fully oriented toward these goals. First, though, the challenge lies in the implementation and scaling-up of HTA capacities in a structured and formalized manner for the benefit of the population, as also for sustainable healthcare provisions. A recent study showed that many African countries are unable to meet the basic requirements for good healthcare systems (22). The lack of political engagement leads to poor governance and human resource challenges are linked to ineffective integration of services in healthcare settings with extremely scarce resources. Other healthcare system concerns prevalent in Africa include financial barriers to healthcare services with high rates of out-of-pocket expenditure. Human resources shortages and “brain drainage” from Africa to Europe, the Middle East, and North America further jeopardize better healthcare outcomes.

According to the results of a survey conducted among MENA countries during the ISPOR Dubai 2019 conference, HTA was mainly utilized to support decisions related to pharmaceuticals and far less for other health technologies (23). The importance of extending the scope of HTA to non-pharmaceuticals including prevention programs was highlighted.

A bottom-up strategy, coupled with multi-stakeholder involvement and engagement, will be a valuable building block for scaling up and implementing HTA capacities. Patient organizations (POs), patient advocates, patients, and citizens can contribute by providing patient- and caregiver-oriented feedback, particularly applicable to the topic of nutrition, whether during hospitalization or in general; an additional means of extending education, advocacy, and support services. POs are formally organized nonprofit groups that (a) concern themselves with medical conditions or potential medical conditions and (b) have a mission to help people affected by medical conditions or to help their families (24).

The interpretation and application of our study may be affected by certain limitations. Questions addressing skill sets specific to the implementation of HTA for nutritional intervention would have benefited the research. Furthermore, an increase in the number of respondents from different countries and stakeholder groups would better represent the variability and inequality of nutritional interventions.

## Conclusion

The early experiences from a few countries show a possible way forward for other African countries in HTA capacity building and implementation in line with the context of specific national healthcare settings and various societal health determinants. Compared to other LMICs, data on the cost-effectiveness of HMN management in African countries is lacking. The prospective multi-country study would be the first of its kind and open perspectives for collaboration with other LMICs in the region. Furthermore, the study will provide valuable insight into the potential of low-cost primary prevention programs while contributing to the recognition of HTA as an ultimate key element of optimal healthcare decision making and resource allocation in LMICs.

## Supplementary material

For supplementary material accompanying this paper visit https://doi.org/10.1017/S0266462323000016. click here to view supplementary material
