# Supplementary material for: Scaling up health technology assessment capacities in selected African countries – A conceivable route ahead
Source: Int J Technol Assess Health Care. 2023 Jan 30;39(1):e9. doi: 10.1017/S0266462323000016 (PMC11574544; doi:10.1017/S0266462323000016)
Supplement: Supplementary file 1 [file S0266462323000016sup001.docx]

**Supplementary material**

Supplementary Table 1 – Questionnaire

1. **Contact Information**
2. Name (first, last):

_________________________

1. Name of organization:

*_________________________*

1. Country:

*_________________________*

1. E-Mail:

*_________________________*

1. **Please describe your health system**
2. What is the structure of your health system?
3. Do you have public insurance coverage? What does it cover? If no, how are health services paid for?
4. What are your government’s priority areas for health? For example, maternal/child health, malaria, cardiovascular diseases.
5. How are health services delivered? Hospitals? Clinics? What geographic areas do these serve?
6. How are health services distributed for preventive/curative measures?
7. What proportion of the health budget spent on hospitals? Different health technologies – pharmaceuticals, medical devices & equipment, consumables, etc
8. How are health service providers remunerated? Do they differ across health professionals, doctors, nurses, etc.?
9. How is procurement organized? Who does the procurement?
10. For UHC, what are the pillars of services within the country?
11. Are there patient organizations in your country? How well organized are they? Are they involved in the health system in any way?
12. **What are the challenges pertaining to HTA?**
13. What are the main challenges in your health system and how do you think these challenges can be addressed by HTA?
14. What are the main challenges in your health system in implementing HTA?
15. **Questions to helps guide development of HTA capacity**
16. What form of HTA is going to be appropriate to this system to address the challenges? (Form: independent agency, support by academic institution, hospital-based)
17. What capacity building activities or other supports are required for the development of system of HTA production and use that will address the challenges in Section C?

Supplementary Table 2 – Section B Questionnaire responses

| Country name/ Questions covered | BQ1. What is the structure of the HS? What is the health budget? | BQ3. What are the (government’s) health priorities? | BQ4 How are health services delivered? | BQ5 How are health services distributed for preventive/curative measures? | BQ6 What proportion of the health budget spent on hospitals? | BQ7 How are health service providers remunerated? | BQ8. How is procurement for medicines organized? | BQ9. What are the range of services to be (or are) provided under UHC? |
| --- | --- | --- | --- | --- | --- | --- | --- | --- |
| Cameroon | Centralized  5.5% of GDP on healthcare | Maternal, newborn, child and adolescent health, CDs  Development of specialized health care services and strengthening health system pillars. | The health care system is controlled by the ministry of public health, consisting of several hospitals. Provincial hospitals and district health care centers which are controlled by a provincial public health delegation. | The main health provider is the public sector, and they provide a majority of the population with medications and cheap health services. The private sector provides services in the country through clinics and hospitals in the urban areas. | The government of Cameroon spends approximately about 5.5% of GDP on healthcare to improve the infrastructure and procure new medical equipment | Remuneration differs across health professionals | A National Centre for Essential Drugs Procurement and Medical disposables manages procurement | PBF structure; Microinsurance schemes; Regional Funds for Health Promotion; NHIS |
| Ethiopia | Decentralized  5% of GDP on healthcare | Reproductive, maternal, newborn, child and adolescent health and nutrition will continue to be top priority for the next 5 years | The devolution of power to regional governments has largely resulted in shifting decision-making for public service delivery from the central to regional and district levels | Access to primary health care units through massive expansion of health centers and health posts as well as deployment of low and mid-level health workforce. | Countries are expected to spend 15% of their national GDP on health service. However, in Ethiopia, it is only 5%. | Remuneration differs across health professionals | Many health facilities are able to develop their own list of medicines, quantify their demand and submit annual pharmaceuticals demand for pooled procurement at the national level. | Populations addressed; services covered and equity of access to health services (target population and geographical areas) |
| Kenya | Decentralized  5.2% of GDP on healthcare in 2018 (ref: WB) | NCDs and CDs, include HIV/AIDs, lower respiratory infections, diarrheal diseases, diabetes, hypertension and malaria and TB. | - | Health services are delivered through four tiers and levels of care based on the kind of health facility. Tier one being community, tier two primary care, tier three secondary referral, tier four tertiary. A number of Tier one to three are available in each of the 47 counties of the country except for Tier 4 facilities that are mainly located in major towns and cities. | Health financing collects funds from taxation, National hospital fund (NHIF), private insurances, employer schemes. Community Based Health Financing (CBHF), out of pocket expenses, development partners and Non-Governmental Organizations. | Health service providers from the public sector are remunerated based upon levels of education attained, years of experience and type of specialization. The amount varies from one cadre to the other in the health sector based on the job groups | Kenya Medical Supplies Authority (KEMSA) is a state corporation under the MoH mandated to procure pharmaceuticals for public facilities. | Access to safe, effective, quality and affordable essential medical products; access to quality essential health services; financial risk protection (Ref: Strategic Investment Plan of 2019-2022) |
| Malawi | Centralized to 3 health care levels | Maternal, Newborn, Child and Adolescent health, Malaria, HIV/AIDS, Tuberculosis, Diarrheal diseases and Cholera, NCDs, other communicable diseases endemic to Malawi | Nearly all formal health care services in Malawi are provided by the Ministry of Health (60% of the services), the Christian Health Association of Malawi (CHAM)(37%) and the Ministry of Local Government (1%). Other providers, namely private practitioners, commercial companies, army and police provide 2% of health services. Primary level: services are delivered through rural hospitals, health centres, health posts, outreach clinics and community initiatives. Secondary level: includes district hospitals and CHAM hospitals. Some of these have limited specialist functions. Tertiary level: at present, tertiary level hospitals provide services similar to those at secondary level, along with a small range of specialist surgical and medical interventions | Using the Essential Health Services: An Essential Package of Health Services (EPHS) is the package of services that the government is providing or is aspiring to provide to its citizens in an equitable manner | Refer to Global health expenditures https://apps.who.int/nha/database/country_profile/Index/en | Remuneration differs across health professionals, however, this responsibility was initially centralized and conducted by the Ministry of health ,but now the government is rolling out decentralization and transferring power to district councils | There is a National Centre for Essential Drugs Procurement and Medical disposable |  |
| Nigeria | Centralized to 3 levels of care  3.95% of the 2018 budget | Curative mainly, less emphasis on investment in preventive. No particular emphasis but basics of provision try to cover treatment of malaria, HIV/AIDs, TB, Hypertension, Diabetes mellitus, early antenatal care, and routine immunization | 3 levels of health care delivery exist: Primary, secondary and tertiary. The health care delivery system in Nigeria operates at three levels, namely primary | - | Only 3.95% of the 2018 budget in Nigeria went to health. The allocation to the Federal Ministry of Health in the 2018 appropriation bill is 340.456bn, out of the total budget of 8.612trillion. | Only 3.95% of the 2018 budget in Nigeria went to health. The allocation to the Federal Ministry of Health in the 2018 appropriation bill is 340.456bn, out of the total budget of 8.612trillion. | Health care delivery is managed at each level – primary, secondary and tertiary. | - |
| Senegal | Decentralized  3.98% of the 2018 budget | Maternal and child health, CDs and NCDs (diabetes, cancers, CVDs, etc.), and nutrition. | Health services are delivered based on the MOHSA’s health map which defines, organizes, and manages the different packages of health care related to each level of the health pyramid. | The health care package is defined for each type of health structure, and all health care package are complementary from the district level to the national hospital level. | - | Health professionals (doctors, nurses, midwives, etc) receive salaries if they are hired by the Government. If not, they are directly paid by their health structures. | A national structure, the Pharmacie Nationale d’Approvisionnement (PNA) is in charge of the national procurement and storage management. PNA is also responsible to distribute the drugs to all public health facilities through its regional agencies, within the country. A partnership exists between PNA and the private health sector which is authorized to command some drugs there. | Pillar 1: Improve the Quality of health services, Pillar 2: Protection against the financial risk, Pillar 3: Decrease the exposure of risk related to the environmental and social determinant of health. |
| South Africa | Decentralized  Health as a percentage of consolidated government budget in 2017/2018 was 13,8%. PHC was 30,8% of the 13.8% while personnel were 61,5%. | Increase of life expectancy, decrease of maternal and child mortality, combating HIV/AIDS, decrease burden of disease from TB, strengthening health system effectiveness | Primary health care services:1) Home and community-based care, 2) Primary care services, 3) Intermediate care. Acute Hospital services:1) District hospitals (first line), 2) Regional hospitals (referral), 3) Tertiary hospitals/Central hospitals with sub-specialist care. | Clinics may or may not have preventative education with students or NGO's. Most services are only curative. | UNICEF document: Per capita spending on consolidated national and provincial health in 2016 was R3155. Health as a percentage of consolidated government budget in 2017/2018 was 13,8%. Primary health care was 30,8% of the 13.8% while personnel was 61,5%. | Remunerations differ greatly between health professionals with allied health workers and nursing sisters being remunerated very little while medical doctors are receiving high remuneration. | The healthcare system comprises both public and private sector. The procurement of pharmaceutical and supplies is undertaken through an open centralized tender system. The NDoH enters into contracts with the pharmaceutical suppliers on behalf of the provinces. | Improving access to quality medical products, improve the efficiency of public sector financial management systems and processes, strengthen the governance and leadership to improve oversight, accountability and health system performance at all levels. |
| Tanzania | Decentralized  3.63% of GDP | Vaccination, nutrition and environmental hygiene, medicine and medical supplies, maternal and child health | In different level of the health facilities there are different clinics which are differed from the primary level to the tertiary level whereby most of time in the primary health facilities there are maternal and child health clinics, CTC clinics, TB clinics while in the secondary and tertiary level they almost cover all other clinics for both communicable and non-communicable diseases and the occupational therapy clinics. | All services are provided at health facilities at the same roof-no distribution for the preventive and curative hence there are different levels deals with health promotion, prevention, curative and rehabilitation services | Refer to medium term expenditure framework (found at ministry of health offices) | Monthly payment for the health care workers with different scale | All procurement is done at health facilities through Medical Store Department (MSD) | Pillar one: Build political support and grassroots demand for UHC at the country level to motivate policies and investments that aim to leave no one behind.  Pillar two: Develop national action plans, define measurable results and celebrate steps forward.  Pillar three: Support a broad, inclusive and cohesive advocacy community to maximise reach, coordination and impact of UHC advocacy. |
| Zimbabwe | Centralized to 3 levels of care  Between 10-15% of the national budget. | Maternal and childcare, HIV/AIDs and TB. | The country is divided into Provinces, each province has different districts; In some provinces you will find a provincial hospital which handles complicated cases, they refer to the Central hospitals if they fail to handle a case.  There are also districts hospitals which provide secondary level of care and follow-up, they will be placed within the community. | District hospitals offer preventive care  Provincial hospitals offer both preventive and curative care. Central Hospitals offer curative care | Between 10-15% of the national budget. Usually falls far short of what is needed. | Health professionals working for the small community clinics are paid by the City Municipality.  Health workers at provincial and central level are paid by the Ministry of Health. | Procurement is decentralized; each facility is allocated a budget from the Ministry of Health and purchase their own requirements. There is a national pharmaceutical company, which procures essential medicines for the whole country and distributes. | - |

CBHI - Community-based health insurance

CDs – Communicable diseases

CVDs – Cardiovascular diseases

MoH – Ministry of Health

NCDs – non-Communicable diseases

NDoH – National Department of Health

NHIS – National Health Information System

OOP – out-of-pocket

TB – tuberculosis

Supplementary Table 3 Community-based health insurance (CBHI) schemes (covering Part A – BQuestion 2: Do you have public insurance coverage? What does it cover? If no, how are health services paid for?)

| **Countries** | **Health insurance coverage** |
| --- | --- |
| Cameroon | Payments are mostly out of pocket  No public insurance coverage |
| Ethiopia | CBHI primarily covers essential health service packages at the health central level. The benefits include both inpatient and outpatient services. A study carried out in 2018 showed that all rural households are not enrolled in CBHI.^*^ CBHI is a targeted subsidy to the poor households. In Ethiopia the CBHI scheme payment per year to enrol in CBHI is less than $10 and therefore even affordable to the poorest. The study shows that utilization of health services among insured households in CBHI was higher. To enhance community health insurance enrolment, it is important to consider factors like travel time to the nearest health institution, perceived quality of care and healthcare cost. |
| Kenya | Public are covered under the National Hospital Insurance fund. The fund generates cash through statutory deductions from registered members aged 18 years and above who earn at least 1000 Kenya shilling monthly. |
| Malawi | Public health care service is free, any additional service is paid out of pocket. However, those that have private insurance cover from various insurance companies seek health care from private hospitals that allow the insurance covers |
| Nigeria | The National Health Insurance Scheme which is currently mainly available for government workers. |
| Senegal | For state employees, the public health insurance covers 80% of the direct health care cost. For community health insurance, there is the same rate of coverage if the health care is provided by a public health structure. If not, the rate is 50%. |
| South Africa | 48.3 percent of healthcare expenditure was from public, 49.8 percent from private sources, and 1.9 percent from donors (2015). Public (serving 84 percent of population) and private health insurance (serving 16 percent) system are separate. |
| Tanzania | Two types of public health insurance, National Health Insurance Fund (NHIF) and Improved Community Health Fund (ICHF) are available. For the ICHF benefit packages are not clear. The NHIF has an attractive benefits package that is offered to its beneficiaries through accredited health facilities countrywide. This package has a total of eleven benefits that are offered to beneficiaries as per Standard Treatment Guidelines issued by the Ministry of Health. |
| Tunisia | Health care providers have complete freedom of prescription. They also have the right to set up in the private sector while remaining under agreement with the publicly run compulsory insurance scheme (CNAM) to facilitate access to care for affiliated patients. Since the national compulsory scheme is publicly run, the government sets the list of covered interventions and the reimbursement rates in the two sectors. |
| Zimbabwe | There are different private insurance companies, and they pay for treatment in either public hospitals or the private hospitals. Treatment coverage depends on the type of cover the patient has. |

^*^Atnafu DD, Tilahun H, Alemu YM. Community-based health insurance and healthcare service utilisation, North-West, Ethiopia: a comparative, crosssectional study. BMJ Open 2018;8:e019613
